# Improving research transparency with individualized report cards: A feasibility study in clinical trials at a large university medical center

**DOI:** 10.1186/s12874-025-02482-9

**Published:** 2025-02-13

**Authors:** Delwen L. Franzen, Maia Salholz-Hillel, Stephanie Müller-Ohlraun, Daniel Strech

**Affiliations:** Berlin Institute of Health at Charité – Universitätsmedizin Berlin, QUEST Center for Responsible Research, Charitéplatz 1, 10117 Berlin, Germany

**Keywords:** Research transparency, Tools, Feedback, Clinical trials

## Abstract

**Background:**

Research transparency is crucial for ensuring the relevance, integrity, and reliability of scientific findings. However, previous work indicates room for improvement across transparency practices. The primary objective of this study was to develop an extensible tool to provide individualized feedback and guidance for improved transparency across phases of a study. Our secondary objective was to assess the feasibility of implementing this tool to improve transparency in clinical trials.

**Methods:**

We developed study-level “report cards” that combine tailored feedback and guidance to investigators across several transparency practices, including prospective registration, availability of summary results, and open access publication. The report cards were generated through an automated pipeline for scalability. We also developed an infosheet to summarize relevant laws, guidelines, and resources relating to transparency. To assess the feasibility of using these tools to improve transparency, we conducted a single-arm intervention study at Berlin’s university medical center, the Charité – Universitätsmedizin Berlin. Investigators (*n* = 92) of 155 clinical trials were sent individualized report cards and the infosheet, and surveyed to assess their perceived usefulness. We also evaluated included trials for improvements in transparency following the intervention.

**Results:**

Survey responses indicated general appreciation for the report cards and infosheet, with a majority of participants finding them helpful to build awareness of the transparency of their trial and transparency requirements. However, improvement on transparency practices was minimal and largely limited to linking publications in registries. Investigators also commented on various challenges associated with implementing transparency, including a lack of clarity around best practices and institutional hurdles.

**Conclusions:**

This study demonstrates the potential of developing and using tools, such as report cards, to provide individualized feedback at scale to investigators on the transparency of their study. While these tools were positively received by investigators, the limited improvement in transparency practices suggests that awareness alone is likely not sufficient to drive improvement. Future research and implementation efforts may adapt the tools to further practices or research areas, and explore integrated approaches that combine the report cards with incentives and institutional support to effectively strengthen transparency in research.

**Supplementary Information:**

The online version contains supplementary material available at 10.1186/s12874-025-02482-9.

## Introduction

The inability to reproduce scientific findings [1–4] and the non- or incomplete reporting of research results [5–9] have raised concerns about research waste, and highlighted the need to promote responsible research practices as a means to increase research value. Transparency is core to responsible research: transparency practices allow the quality of research to be appraised, mitigate biases, discourage poor methodological practices, and facilitate comprehensive evidence synthesis [10–13]. The relevance of these practices is emphasized in initiatives to reform research assessment [14, 15], which have included calls to increase recognition of researchers who commit to robust and transparent practices [16]. One of the principles underlying the 2022 Agreement on Reforming Research Assessment is to focus research assessment criteria on quality, where “research is carried out through transparent research processes and methodologies” [17].

Reforming how research is performed and assessed requires evaluation and communication about the current uptake of responsible research practices, including transparency. Such monitoring efforts largely focus on a single phase of the research process (e.g., results reporting) and communicate findings at a group level, such as institution or country. For example, the EU Trials Tracker shows compliance of clinical trials with results reporting requirements and presents these data as a dashboard at the level of institutional sponsors [18]. In turn, the French Open Science Monitor shows the evolution at the national level of several open science practices, such as open access and mentions of data sharing in publications [19]. These initiatives and others [20–23] provide baseline information against which change can be measured and empower efforts to drive research transparency.

Policy and monitoring activities reflect top-down approaches to inform and trigger change in research transparency. Yet improvement ultimately requires researchers to take specific actions, such as report results and methods in a timely manner and in sufficient detail. Thus, effective research reform also requires bottom-up approaches to empower researchers through feedback and guidance on transparency at the level of individual studies. Efforts to increase the transparency of individual studies largely focus on publications. Journals have experimented with awarding authors open science badges to incentivize practices such as open data [24, 25]. The ScreenIT pipeline screens preprints in medRvix and bioRxiv for common problems in how research is reported and provides rapid feedback to help authors improve transparency and reproducibility [26].

Building on these efforts, we aimed for a complementary approach to provide feedback and guidance to investigators on transparency practices associated with different phases of a study. While such a feedback tool would be beneficial across research areas and study types, our feasibility study focused on clinical trials. Clinical trials are well suited to evaluate the feasibility and usefulness of such a tool for both normative and technical reasons. The significance of clinical trials to patient health has inspired ethical guidelines and legal requirements for transparency practices across the research lifecycle, from prospective registration to timely results in registries and journal articles [27–29]. In turn, clinical trial registries provide a ready list of studies and collect data relating to those trials, from study start to completion, which enables evaluation of these practices [30–32]. In previous work, we developed tools to monitor and communicate performance on these practices at the level of institutions, which could be adapted to the level of individual studies [21].

This study’s primary objective was to develop a scalable tool to provide feedback and guidance on transparency across research phases of an individual study. We designed individualized “report cards” that summarize the performance of a given clinical trial across several transparency practices and provide tailored guidance for improvement. We also created an infosheet with an overview of recommended transparency practices and relevant guidelines and regulations.

The secondary objective was to investigate the feasibility of implementing these tools to improve trial transparency. We conducted an exploratory, single-arm intervention and disseminated individualized trial transparency report cards and the infosheet to investigators at the Charité – Universitätsmedizin Berlin, in collaboration with the institutional Clinical Study Center. We surveyed investigators on the self-reported usefulness of the materials and evaluated the included trials for improvements in transparency.

## Results

### Tools: report cards and infosheet

**Fig. 1:**
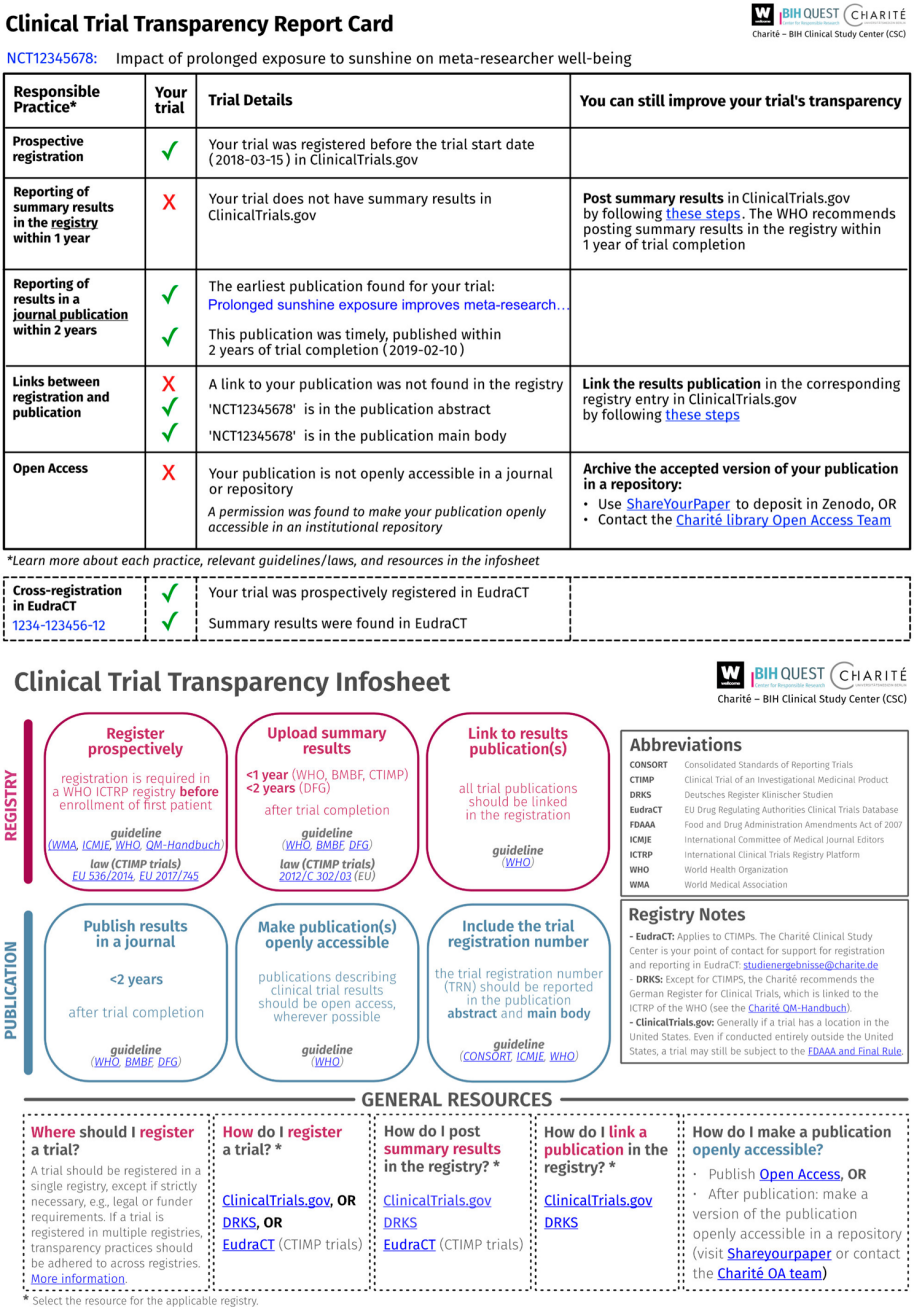
Clinical trial transparency tools: report card and infosheet. Example report card with individualized feedback and guidance on the transparency of a trial. A mock trial is shown for illustrative purposes. Infosheet summarizing relevant laws, guidelines, and best practices related to clinical trial registration and reporting. The infosheet is tailored to the institutional (Charité) and national (Germany) context

We developed study-level report cards that combine individualized feedback and guidance on the transparency of a given trial (Fig. 1). The report cards provide feedback on the following transparency practices: (a) prospective registration, (b) availability and timeliness of summary results in the registry, (c) availability and timeliness of a results publication (earliest if multiple), and if a publication exists, (d) links between registration and the results publication, and (e) open access status of the publication. In case of a validated cross-registration in the EU Clinical Trials Register (EUCTR), the report card additionally displays the following practices based on the EUCTR registration: (a) prospective registration, and (b) availability of summary results in the registry. For transparency practices that can still be improved after trial completion, the report card includes a call-to-action and a link to resources to implement the practice. For reporting of summary results and publication linking in the registry, the report card points to instructions to complete the practice on the relevant registry. For closed-access publications, the report card provides instructions on how to archive a version of the publication in an openly accessible repository.

We also developed an infosheet with an overview of guidelines and laws relating to clinical trial transparency, tailored to the national and institutional contexts (Fig. 1). The infosheet summarizes transparency practices relating to trial registration and reporting, their normative basis, and available resources to implement each practice.

The final versions of the report card and infosheet incorporated feedback from expert reviews and cognitive interviews. Suggestions for improvement from participants largely related to the visual design, scope, and implementation of the tools. Themes are elaborated in Supplement 1 Table 1.

### Feasibility study

#### Sample characteristics

**Fig. 2:**
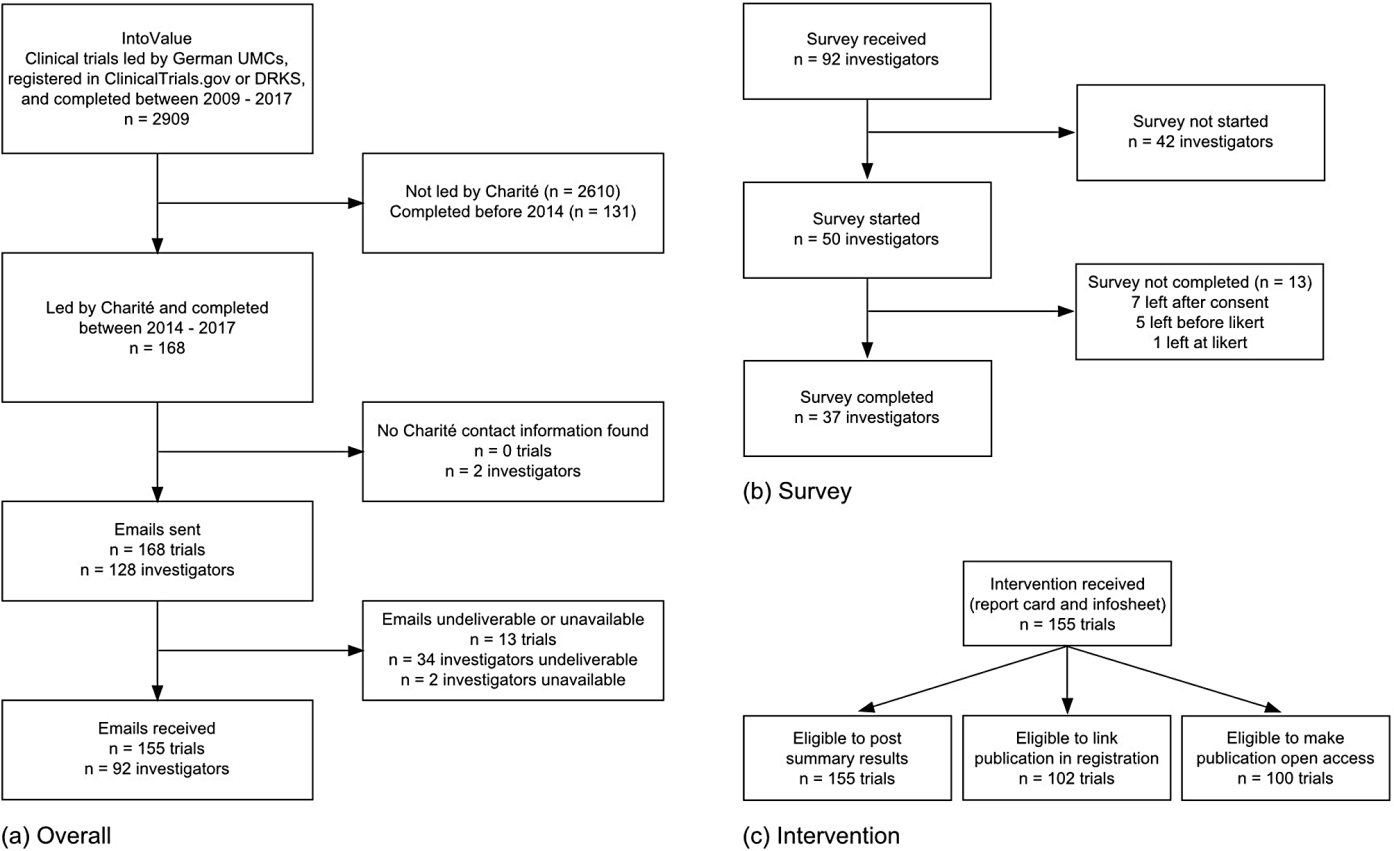
Screening flowchart of trials and trial investigators. (**a**) shows overall screening. (**b**) shows screening of investigators for the survey analysis. (**c**) shows screening of trials for the analysis of improvement in transparency practices; eligible trials include trials that had already completed the practice at intervention launch

To investigate the feasibility of using report cards and an infosheet to improve trial transparency, we started with a dataset of 2,909 trials generated in previous studies (known as “IntoValue”; see details in the Methods). After limiting this set to trials led by the Charité and completed between 2014 and 2017, we contacted 128 investigators responsible for 168 trials. After excluding unreachable investigators, our analysis sample included 92 investigators responsible for 155 trials. Figure 2 (a) details screening of both trials and investigators. Characteristics of the included trials and investigators are in Supplement 2 Table 1.

### Survey

A total of 37 researchers (40% of the 92 contacted) completed the survey in the 12 weeks between survey launch on 25 May 2022 and study close on 17 August 2022. Figure 2 (b) shows a flowchart of investigators and exclusion from analysis at each stage. Supplement 3 Fig. 1 shows the response timeline across the survey fielding period. The survey took respondents generally just under four minutes, with a median response time of 237 s (Interquartile Range: 124–402). The majority of respondents self-reported as study leads or study doctors (*n* = 32, 86%); see Supplement 3 Fig. 2 for additional details on respondent roles.

**Fig. 3:**
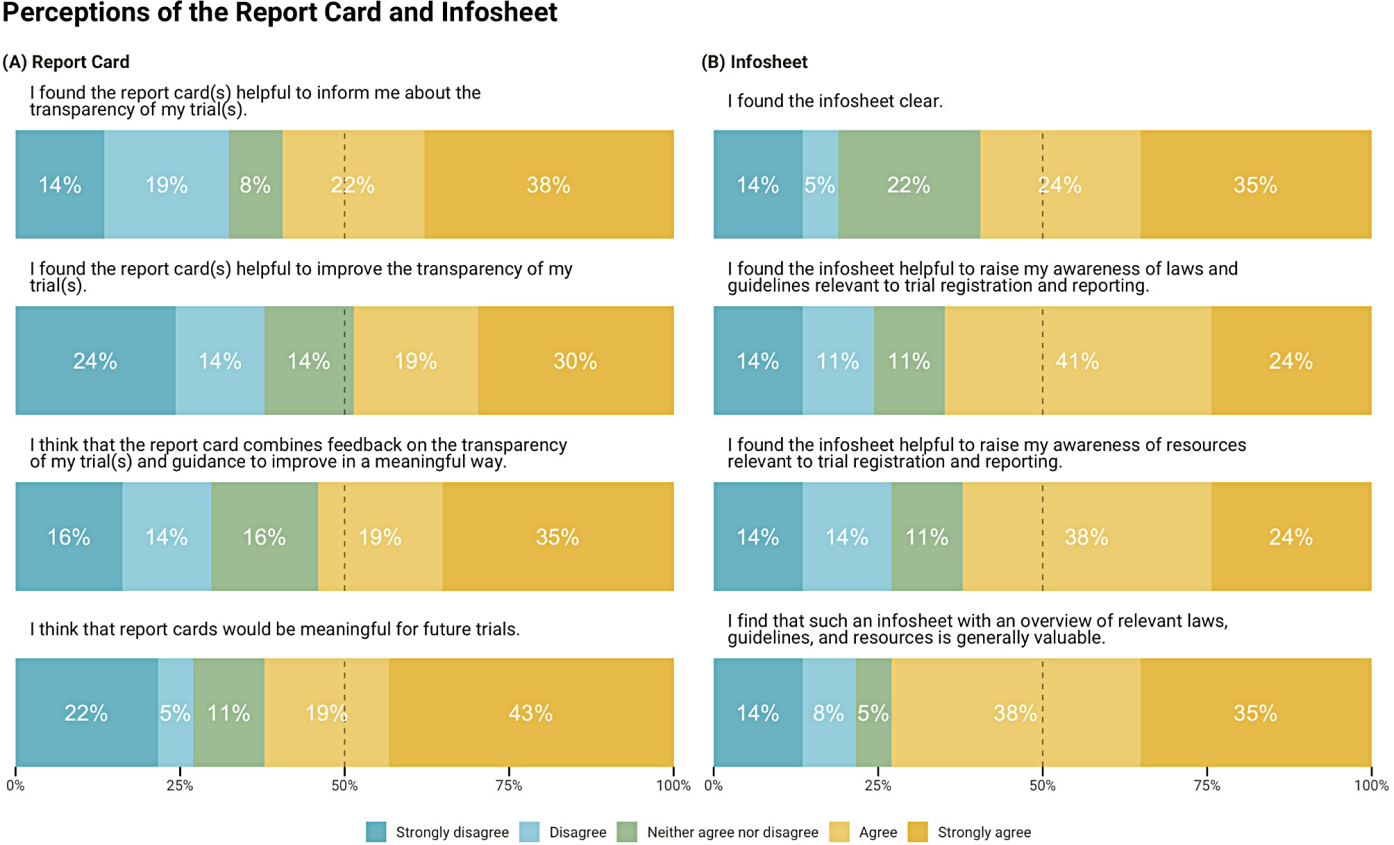
Perceptions of the (**a**) report card and (**b**) infosheet

The survey asked trial investigators to rate their agreement with each of eight statements about the report card and infosheet (for example, “The infosheet is clear.”). Respondents shared overall positive perceptions about the report card and infosheet, with at least 49% agreeing or strongly agreeing with all provided statements, and mean scores on all individual items ranging from 3.2 to 3.7, on a scale from 1 to 5. A peak of 73% of respondents found the infosheet with an overview of relevant laws, guidelines, and resources generally valuable. The statement with the lowest agreement (49%) and highest disagreement (38%) was whether the report cards were helpful to improve their own trial’s transparency. Figure 3 shows responses to each item; means with 95% confidence intervals are presented in Supplement 3 Fig. 3.

A subgroup analysis of three participants who reported not reviewing the materials before starting the survey showed that they had all selected the most negative option (i.e., strongly disagree) across all statements on both the report card and infosheet. Another subgroup analysis showed no meaningful difference between study leads and doctors (*n* = 32) versus other respondents (*n* = 5). See Supplement 3 for further details on these subgroup analyses.

Respondents were invited to share comments on the materials. After removing non-substantive responses (e.g., “I have no comment”), 17 remained. Comments included positive remarks and suggestions for improvement of the materials, as well as broader challenges around the management and coordination of clinical trials. Supplement 3 Table 1 details the suggestions and challenges provided in comments.

Investigators also had the opportunity to provide corrections to their report card. In our manual review of the 18 provided corrections, we found that only five were valid, while the remaining were incorrect understandings of the practices or additional information on the trials beyond the scope of the report card. See Supplement 3 for details.

### Improvement in transparency practices

**Fig. 4:**
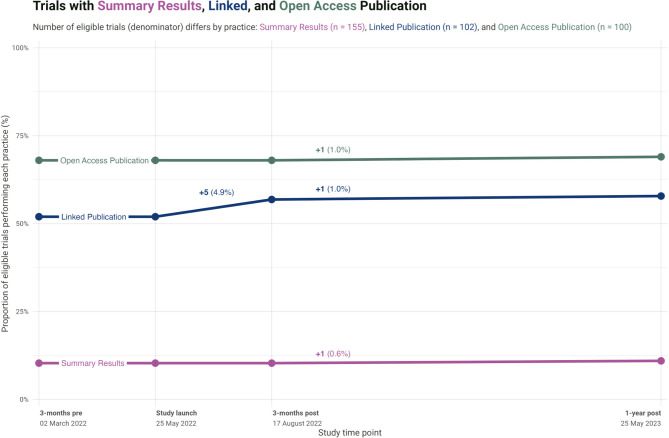
Overall proportion of trials performing each practice at each study time point (three months prior to study launch, at study launch, and after study launch at three and 12 months). The different denominators across practices reflect distinct requirements to perform each practice. Summary results: denominator is all trials (*n* = 155). Linked publication: denominator is all trials with a publication (*n* = 102). Open access publication: denominator is all trials with a publication and a publisher permission to self-archive the paper in an institutional repository (*n* = 100)

Trial transparency improved minimally across the follow-up period for eligible trials (see Fig. 2 (c) for a screening flowchart of eligible trials). The largest improvement was in linked publications, which increased 5.9%: five trials added links to their publications in the registry in the three months following our intervention, and one additional trial did so within one year of the intervention. One of these trials also posted summary results in the registry. While these changes were small, no changes were seen in the three months preceding the intervention (Fig. 4). Additionally, one publication became openly accessible on the journal website and not a repository, suggesting this was not triggered by an investigator and was hence independent of our intervention.

## Methods

### Tools: report cards and infosheet

#### Design

We set out to develop report cards with individualized feedback on the transparency of a given trial. The report cards aimed to: (a) provide feedback on registration and reporting best practices at the level of an individual trial, (b) indicate visually (e.g., tick mark, cross) whether a practice was followed, (c) include the trial details relevant to each practice, (d) be succinct (page), and (e) be generated with open source code for scalability. We also designed an infosheet that provides an overview of guidelines and laws relating to clinical trial transparency. The infosheet aimed to: (a) provide an overview of the recommended or required transparency practices and their normative basis (i.e., guidelines or laws), (b) include links to resources for each practice, (c) be tailored to the German and institutional context, and (d) be succinct (one page). The design of the report cards and infosheet was shaped by feedback obtained in cognitive interviews and from experts in clinical trial transparency Supplement 1. The report cards and infosheet were presented in English.

### Trial transparency dataset

The report cards require a structured trial transparency dataset, i.e., a dataset of clinical trials and their performance across several transparency practices. We previously developed methods to assess transparency practices for a cohort of trials using a combination of automated and manual approaches, which are described in detail elsewhere [21, 33–35]. The dataset is based on publicly available data from registries and publications and does not include information on trial participants or individual patient data. Supplement 4 summarizes the main steps to prepare a trial transparency dataset for use with the report cards.

### Technical implementation: translating the dataset into study-level report cards

We developed a pipeline to automatically generate individualized report cards based on the trial transparency dataset (see Supplement 4 Fig. 1). In the first step, we designed a template of the report card in Affinity Designer, a vector graphics editor. The report card template is a visual representation of a trial’s performance across transparency practices. Since trials perform differently across transparency practices, the report card template includes all possible outcomes of a trial’s performance represented as layers. The report card template was exported as Scalable Vector Graphics (SVG), which is an image format based on Extensible Markup Language (XML). This allows the report card template to be accessed and modified using code. In the second step, report cards for each trial were assembled automatically using a custom-made Python script that selected the correct layers to include for each trial based on the trial transparency dataset. In the third step, each report card was automatically exported as a PDF for dissemination to study participants. An example report card is displayed in Fig. 1. The infosheet (Fig. 1) was generated in Affinity Designer and Affinity Publisher and adapted on an iterative basis.

### Feasibility study

We conducted a feasibility study to pilot the use of the tools at the Charité – Universitätsmedizin Berlin, in collaboration with the institutional Clinical Study Center. The study used a cross-sectional, interventional design and included a descriptive analysis of two main outcomes: self-reported evaluation of the tools via a survey, and improvement in transparency practices following the intervention of the tools. All trials received the intervention (single-arm), as the number of trials eligible to be included in our analysis was too small for a control group and sufficient power to detect a minimum effect size of interest.

### Ethical approval and study protocol

This study was approved by the ethics review board of the Charité – Universitätsmedizin Berlin (#EA1/337/21). The study protocol was preregistered and is openly available in the Open Science Framework (OSF) at: 10.17605/osf.io/stnp5. Deviations to the protocol are outlined in OSF: https://osf.io/wxayg.

### Sample development

This study used a cohort of trials and associated investigators. Screening criteria were assessed for both trials and investigators, in part prior to study launch and in part prior to analysis. Criteria are outlined below and detailed in Supplement 2 Table 2.

#### Trials

This study draws from two cohorts of registered clinical trials and associated results, referred to as “IntoValue” [36]. The IntoValue dataset consists of clinical trials led by a German University Medical Center (i.e., as either sponsor, responsible party, or host of the principal investigator), registered in ClinicalTrials.gov or the German Clinical Trials Register (DRKS), and considered as complete per the registry between 2009 and 2017. For this feasibility study, the IntoValue dataset was limited to trials led by the Charité with a completion date between 2014 and 2017 (i.e., the second IntoValue cohort); these more recently completed trials were selected, given the higher likelihood of reaching investigators and increased feasibility of implementing practices. To reflect the most up-to-date status of trials, we downloaded updated registry data for the trials in this cohort on 19 May 2022 and reapplied the original IntoValue exclusion criteria: study completion date before 2014 or after 2017, not considered as complete based on study status, and not interventional. Trials were excluded if no Charité investigators were found or reached.

#### Trial investigators

We developed a list of investigators for our sample of trials based on contact information retrieved from clinical trial registries. We used the following registry fields: from ClinicalTrials.gov, responsible party (if this included principal investigator), and overall study officials (principal investigator, study director, study chair); from DRKS, primary sponsor, and contacts for scientific and public queries. As contact information is often removed from registrations after trial completion [37, 38], we used the R package cthist [39] to retrieve the current (as of 19 May 2022) as well as all historical versions of registrations. If no contact information was available in the registry, we searched Google and the Charité address book using the investigator’s name in the registry and the Charité email pattern, as well as trial details such as trial registration number and title keywords as needed. If multiple investigators or multiple emails for the same investigator were available, all were used; if the registration provided an email for the project or research group, this was included in addition to the investigator email(s). Investigators were excluded if no contact information was found, and trials were excluded if no contact information for any trial investigator was found. Per our preregistration, after the email-based intervention, investigators were excluded if all emails were undeliverable or if auto-replies indicated extended leave throughout our study timeframe; trials were then excluded if no trial investigator remained.

### Materials

#### Transparency practices and report cards

In line with the requirements for the report cards (see “Trial transparency dataset”), the IntoValue trial cohort includes data on transparency practices. Data obtained through automated methods were updated at several time points during the study. Registry data for ClinicalTrials.gov was queried from the Aggregate Analysis of ClinicalTrials.gov (AACT) database [40], and DRKS was webscraped prior to the start of the intervention and at the end of follow-up. At study end, we additionally used cthist [39] to get historical versions of registrations in order to complete the analysis of each study time point. To determine the open access status, we used UnpaywallR [41] to query the Unpaywall [42] Application Programming Interface (API) at each study time point; to determine publisher permissions for self-archiving, we queried the Shareyourpaper API (using a custom-made Python script [35]) at study launch. Additional manual validations were done for the open access status of publications as well as for cross-registration in the EUCTR. Protocols for these validations were prospectively uploaded on OSF (https://osf.io/3upgy and https://osf.io/mjpzh), and methods are summarized in Supplement 5. The resulting trial transparency dataset was used to generate the report cards. After study launch, we found a bug in the code and subsequently made six amendments to the dataset and report cards, namely we added: one cross-registration in EUCTR, four publication links in DRKS, and one summary result in DRKS. More detailed information can be found in the project repository in GitHub: https://github.com/quest-bih/trackvalue.

#### Questionnaire development and pretesting

We conducted an anonymous survey to assess the usefulness of the report cards and infosheet. The survey was generated in LimeSurvey and hosted on a server at the QUEST Center for Responsible Research at the Berlin Institute of Health at Charité – Universitätsmedizin Berlin. After informed consent and prior to starting the survey, respondents were asked whether they had reviewed the materials, and if not, they were prompted to do so and return to the survey. Respondents were asked to evaluate the report card and infosheet using a 5-point Likert scale across four items for each tool. Respondents were also asked to indicate their role(s) in the trial, and were invited to provide corrections to the report card(s), if applicable, as well as general comments. The survey was presented in German. The design of the survey was shaped by feedback obtained in cognitive interviews (see Supplement 1) [43]. The survey is provided in OSF (https://osf.io/5z463).

#### Administration of the intervention and survey

Investigators were sent an invitation email including: a letter (co-signed by the Charité Clinical Study Center) listing all their trials, a report card for each trial they led, and an infosheet. The email and letter also included an invitation to participate in the anonymous survey as well as a notice that the transparency of their trials would be re-evaluated three months after the first email (see a sample invitation in OSF: https://osf.io/9qkdp). Emails were sent from a functional mailbox created for the study that was regularly monitored over the course of the study. Any auto-replies indicating undeliverable emails or extended leave were preserved. Follow-up emails were sent at weeks one, five, and 11. As the survey was anonymous, and we did not know who had already completed it, all reminders were sent to all investigators included in the study. We reviewed the survey responses on an ongoing basis to identify any valid corrections to the report cards. For valid corrections, we updated our dataset and generated and sent amended report cards to all investigators associated with the trial.

### Analysis

#### Analysis of perceptions of the report card and infosheet

We describe investigators’ perceptions of the report card and infosheet per the survey. Our primary outcome is how much respondents agree with four positive statements about each tool. Statements (for example, “The infosheet is clear.”) were presented with a 5-point Likert-type response scale, with options ranging from 1 = strongly disagree, to 5 = strongly agree. We report the proportion and means with 95% confidence intervals of each Likert response. Survey responses were considered complete if all Likert items were completed, and as all Likert items were required, we did not evaluate missingness. We report the survey response rate, the response timeline across the survey fielding period, and exclusion from analysis at each stage (e.g., due to undeliverable emails). As the survey was anonymous, we could not evaluate non-response error or adjust for possible non-representativeness of the respondents; we also could not determine whether unique respondents came from unique visitors. We evaluated response time as a quality check for active participation and to avoid counting bots. We report the number of respondents who reported not reviewing materials before starting the survey and perform a subgroup analysis of these respondents. We also perform a subgroup analysis of study leadership. We report the number of corrections, and evaluate and report the number which are valid; corrections were reflected in subsequent report card versions. We also report the number of comments after removing non-responses and describe common themes identified in respondent feedback. Corrections and comments were reviewed and adjudicated by consensus by two researchers (MSH and DLF).

#### Analysis of improvement in transparency practices

We assessed trials for three transparency practices that could still be changed after trial completion: (1) reporting of summary results in the registry, (2) publication links in the registration, and (3) open access status of publication. The publication-based analyses were limited to trials with publications available at the time of intervention launch; the open access analysis was further limited to trial publications that could be self-archived in an institutional repository based on publisher policies (“green open access”) at the time of intervention launch. We report the overall proportion of trials performing each practice at the following time points relative to our intervention: three months prior to launch, at launch, three months after launch. As a protocol deviation, we additionally report rates a year after the intervention.

### Software, code, and data

All the code generated in this study is openly available under an open license in GitHub: https://github.com/quest-bih/trackvalue. Data collection and analysis was conducted in Python [Version 3.9] and R [Version 4.4.1]. This manuscript was written by interleaving regular prose and analysis code in Quarto [Version 1.5.55]. With the exception of investigator contact information and survey comments and corrections, all the data is openly available in the GitHub repository of the project and in Zenodo: 10.5281/zenodo.10467054.

### Reporting guidelines

This study uses both an intervention and a survey, and follows the CROSS Consensus-Based Checklist for Reporting of Survey Studies [44] and CONSORT for Pilot and Feasibility Trials [45]. Reporting checklists are available in Supplement 6.

## Discussion

We developed scalable, open source tools to provide individualized feedback and guidance on research transparency across phases of a clinical trial. We investigated the feasibility of using these tools to improve clinical trial transparency in a sample of completed trials led by researchers at the Charité – Universitätsmedizin Berlin, one of the largest university hospitals in Europe (https://www.charite.de/en). Overall, researchers found the tools helpful to inform them of the transparency of their trial and to raise awareness of relevant guidelines, laws, and resources. However, improvement across transparency practices one year after dissemination of the tools was limited. This study demonstrates the feasibility of generating and communicating study-level feedback to researchers, and can inform future interventions to improve transparency and the adoption of responsible research practices more broadly.

## Summary of findings

### Perceptions of the report card and infosheet

Survey responses indicated that the report cards and infosheet were generally well received by study participants. The infosheet, in particular, stood out as being a valuable resource, suggesting that succinct visual summaries of what is expected of investigators (based on applicable laws, guidelines, and policies) may be a fruitful approach to build awareness of transparency requirements alongside other forms of training. Participants’ views on the report cards were largely positive. Lower ratings on the usefulness of the report cards to improve trial transparency may reflect the need for additional support or the removal of barriers to implement changes in practice. We attempted to bridge this gap by linking to detailed guidance on how to perform each practice.

### Transparency improvements in practice

Trial transparency improved minimally over the follow-up period, with improvements largely limited to linked publication in the registry. The discrete improvement for this practice, which involves manually adding a link to a results publication in the registration, may reflect the lower time and effort needed compared to other practices, or perceived hurdles associated with summary results reporting (e.g., fear of jeopardizing journal publication [46]) and self-archiving (e.g., fear of copyright infringement [47]). Furthermore, our study called for action on trials reported as complete up to eight years prior to this intervention, which may have been too long ago for these practices to be feasibly addressed. Prospective reminders while a study is ongoing or for recently completed studies, as already implemented in several registries (e.g., ISRCTN [48]), may be more effective in driving change.

Perceived barriers to implementing transparency practices were noted in investigators’ survey responses. While the survey did not aim to solicit feedback beyond the tools, numerous respondents volunteered several challenges (see Supplement 3 Table 1), including lack of clarity around best practices (e.g., applicable guidelines and/or laws in case of multiple registrations of a single trial), diffusion of responsibility (e.g., limited control over transparency practices due to journal guidelines), and obstructive institutional processes and bureaucracy around the conduct of trials, which take time and resources away from other activities. Taken together, investigators’ positive perception of the materials yet limited improvement in transparency practices, as well as unprompted comments on challenges, suggests that implementing research transparency is a multifaceted issue and that interventions likely need to be embedded within systemic changes.

### Research in context

Previous empirical research has explored the impact of interventions at the level of individual studies to increase clinical trial transparency. Maruani and colleagues conducted a randomized controlled trial in 2014 to evaluate the impact of sending email reminders to investigators of completed Phase IV trials about their requirements to post results [49]. At six months following their intervention, results had been posted for 24% of trials that had received the intervention versus 14% in the control group, suggesting that reminders may effectively prompt results reporting. A 2023 study at the German registry DRKS similarly found that email reminders were effective in prompting investigators to complete draft registrations: five weeks after the intervention, 11% of trials that had been sent an email had completed their registrations, compared with 4% of trials that received no communication [50].

Approaches such as report cards or reminders may have greater impact when embedded within a holistic behavior change approach that brings together capability, motivation, and opportunity [51], as suggested by previous work across research, policy, and practice. For example, the launch of the EU Trials Tracker as a public audit tool was complemented by an advocacy campaign, which eventually spurred policymakers and other stakeholders to demand change from institutions [52]. Research performing institutions subsequently rapidly improved their capacities to address these practices, and within a few years, universities’ policies had been strengthened [53] and trial reporting increased from 50% in January 2018 to 84% in September 2023 [54]. Additional external factors further reinforced opportunity: in February 2020, the Make It Public strategy was adopted in the United Kingdom, whereby clinical trials are automatically and centrally registered by the Health Research Authority following ethics approval, which saves researchers this extra step [55].

In the United States, efforts to understand existing support structures for trial registration and reporting [56] were accompanied by improvements in transparency at academic organizations. Institutional programs to support compliance with registration and reporting requirements launched a variety of activities, including audits to identify problem records and bring them into compliance, prospective reminders, policy development, enforcement, standard operating procedures, centralized resources, training, and consultation [57–59]. Following the implementation of such a program at Johns Hopkins University School of Medicine, non-compliance for mandated registration and reporting dropped from 44% (339/774) in 2015 to 2% (32/1,304) in 2020 [57]. More broadly, the emergence of interventions implemented by key stakeholders at different levels offers a rich opportunity to investigate effective, multifaceted strategies for research improvement.

### Strengths, limitations, and challenges

Our study has several strengths. The report cards offer a single resource that brings together individualized feedback and guidance on the implementation of specific practices. The approach to generate the report cards is openly available and can be scaled to other institutions and contexts that have, or are seeking to develop, an overview of their studies and associated practices. Importantly, this approach leverages automated approaches to drive transparency at scale, while not being tied to a specific stakeholder or infrastructure. Moreover, we collaborated with an institutional core facility: this approach may be a strength by increasing institutional buy-in and the likelihood of sustainable adoption of such a tool; however, it may also have shifted investigators’ attention away from the tools towards institutional aspects.

Our study also faces several limitations. The report cards rely on the information in the registry being accurate and up-to-date, which is not always the case [38, 60, 61]. For EUCTR trials, prospective registration was assessed based on dates available in the registry; however, in principle all EUCTR trials should be prospectively registered, as registration is linked to regulatory approval by national competent authorities. The small sample size in this study precluded randomization and a control group with sufficient power to detect a minimum effect size of interest. The design therefore did not allow us to test whether our intervention drove the changes (i.e., causality), which may also have been prompted by researchers themselves or other co-occurring pressures in the German clinical trial landscape [62]. However, as our aim was to test the feasibility of delivering such an intervention, the lessons learned may inform a larger randomized controlled trial. Another limitation of our study is that the invitation letter and survey were provided in German, while the report card(s) and infosheet were provided in English. This may have limited the reach or understandability of the materials. Future studies may consider generating multilingual materials.

We identified several challenges related to material development and the intervention, which may impact the scalability of this type of intervention. Despite the use of automated approaches, additional manual checks were still required to validate the data. Researchers or organizations looking to use or adapt this approach would need time, resources, and skills to create the trial transparency data set (including performing manual checks) as well as to generate the bespoke report cards using the open source code and materials. Another challenge was balancing comprehensiveness and clarity in the infosheet; this could be supported by engaging visual design experts and by using dynamic formats such as HTML instead of static PDFs. Additionally, step-by-step instructions on how to implement transparency practices were not available across all registries at the time of the intervention. Contact information of investigators was not always readily available in the registry, and in some cases required searching historical versions of a trial or internet searches.

### Scaling beyond transparency in clinical trials

The report cards were designed for scalability and extensibility. They may be adapted to provide individualized feedback and guidance on further aspects of transparency or responsible research. These include data sharing, robustness (e.g., randomization), patient-orientedness (e.g. patient engagement in study design), and ethical safeguards (e.g., conflict of interest disclosure). The report cards may also be adapted for use in other types of studies and disciplines. This would require a core set of practices to monitor, an overview of studies, and automated and/or manual approaches to extract, process, and validate the data to include in the report cards [63]. While many study types and disciplines do not yet have formalized best practices for responsible research comparable with those for clinical trials, a core set of practices to monitor within a discipline may be established through community consensus, as done in biomedicine [64]. We expect broader applicability of tools that provide study-level feedback as registration becomes more common across certain fields (e.g., animal research [65]).

### Implications and future work

Future work is needed to further evaluate the use of report cards to address transparency, and iteratively transfer this potential solution to policy and practice. Our feasibility study may inform a larger randomized controlled trial at the level of multiple institutions, and institution-led behavior change interventions more broadly. The larger sample sizes would allow for more robust statistical methods such as regression analyses to explore how trial, researcher, or institutional characteristics relate to improvements in transparency [33]. These studies may consider following up with investigators who implement practices to understand whether the intervention indeed prompted the behavior. Future work may also consider evaluating prospective use of the tools. For example, report cards that prompt investigators before a specific practice is due, or an infosheet disseminated at study start that sets out expectations for researchers before the work is conducted. Report cards may be particularly well suited to help funders monitor and support compliance with grant requirements, which typically span the entire project lifecycle. With the emergence of further studies to improve research transparency and other responsible research practices, evidence synthesis is needed to compare interventions and inform priority setting for future research improvement efforts; see for example ongoing work to build a living evidence summary of interventions to improve reproducibility [66].

Future studies should adopt a systemic approach and consider the role of capability, motivation, and opportunity when designing an intervention to improve transparency [51]. For example, combining the report cards with initiatives that reinforce motivation, such as awards or broader recognition of responsible research practices in research assessment [16], may be an effective approach. Proposed solutions will also need to be tailored to the specific context and resources of a given institution. Engaging with researchers and other stakeholders, who have first-hand experience of challenges and needs, will be key towards identifying actionable areas for improvement to drive sustainable change.

## Electronic supplementary material

Below is the link to the electronic supplementary material.

Supplementary Material 1

## Data Availability

All the code generated in this study is openly available under an open license in GitHub: https://github.com/quest-bih/trackvalue. Data collection and analysis was conducted in Python [Version 3.9] and R [Version 4.3.2]. This manuscript was written by interleaving regular prose and analysis code in Quarto [Version 1.4.549]. With the exception of investigator contact information and survey comments and corrections, all the data is openly available in the GitHub repository of the project and in Zenodo: https://doi.org/10.5281/zenodo.10467054.
