# Supplementary material for: Improving research transparency with individualized report cards: A feasibility study in clinical trials at a large university medical center
Source: BMC Med Res Methodol. 2025 Feb 13;25:37. doi: 10.1186/s12874-025-02482-9 (PMC11823227; doi:10.1186/s12874-025-02482-9)
Supplement: Supplementary file 1 — Supplementary Material 1 [file 12874_2025_2482_MOESM1_ESM.pdf]

SUPPLEMENTS

Improving research transparency with individualized report cards: A feasibility study in clinical trials at a large university medical center

1. Expert reviews and cognitive interviews on the materials and survey
2. Screening criteria and characteristics of trials and investigators
3. Survey administration and detailed results
4. Steps to generate individualized trial transparency report cards
5. Methods for manual validation of open access status of publications and cross-registration in the EUCTR
6. Reporting checklists

1 Expert reviews and cognitive interviews on the materials and survey

Methods

We solicited expert reviews to get feedback on the content and design of the invitation letter, report cards, infosheet, and survey. We contacted content experts, experts in questionnaire development, and experts in statistical analysis [64, Guideline 7.7]. Reviews were unstructured and tailored to each person's expertise. Content experts included researchers and administrators involved in the conduct and management of clinical trials. Colleagues with extensive experience conducting surveys reviewed and consulted on the questionnaire. A statistical consultant at the Charité reviewed the analysis plan prior to preregistration of the protocol.

We also performed think-aloud cognitive interviews to identify challenges with the visual design, wording, and navigation of the materials, and to evaluate whether the respondents interact with the materials and understand the questions as intended by the research team [64, Guideline 7.8]. Participants (n = 3) were identified within our networks and were selected based on previous or ongoing experience conducting or administering clinical trials at the Charité, in order to maximize similarity with the sample population. Participants received an invitation email to participate in a 1-hour interview conducted over Microsoft Teams. One team member led the interview (MSH) while another team member (DLF) observed and made notes of the interview. After informed consent, participants were asked to walk through the invitation letter, report card, infosheet, and survey and think aloud to capture their thought processes when engaging with the study materials. The cognitive interview guide is available in OSF (https://osf.io/xtpjc). Based on the feedback shared in each interview, we modified the materials prior to the following interview.

Results

Themes (Supplement 1 Table 1) for the design of the report card included: provide actionable information by combining feedback and guidance, and by highlighting practices that can still be improved; contextualize feedback and guidance by providing study details and the normative basis for each practice; use clear visuals and accessible language to convey the message quickly. The main theme for the design of the infosheet was balancing comprehensiveness and clarity, by tailoring the information to the audience (e.g., acknowledge institutional guidelines and resources), and by carefully considering the structure and layout. With a few exceptions due to feasibility, the feedback was incorporated into the report cards and infosheet.

Supplement 1 Table 1: Emerging themes for the design of the report cards and infosheet developed in this study.

Theme	Notes
Provide actionable information	• Combine feedback and guidance for improvement in the same place<br>• Limit guidance to practices that can still be improved<br>• Include resources to reduce barriers to taking action (e.g., hyperlink to implement change in registry)<br>• Link to institutional support, where applicable (e.g., core facilities)<br>• Use language to emphasize actionability (e.g., “You can still improve your trial’s transparency”)
Visual representation of performance	• Convey the right message quickly using appropriate symbols and colors<br>• Use colorblind-friendly colors
Contextualize feedback and guidance	• Highlight the normative basis for each practice (e.g., ethical guideline or legal requirement)<br>• Provide study details for each practice to contextualize feedback (e.g., days between trial completion and results publication)<br>• Provide study details in a recognizable way (e.g., consider whether to refer to a publication with a citation or a publication title)
Accessible Language	• Avoid using acronyms. If acronyms are used, check for familiarity with the acronym (e.g., EudraCT or EUCTR to refer to the EU Clinical Trials Register)<br>• Avoid language that may be perceived as established terminology (e.g., “trial score”)<br>• Use simple and concise language<br>• Use specific language (e.g., “earliest publication” if report card only includes information about the earliest publication)<br>• Check for possible misunderstandings

Report card

Theme	Notes
Balance comprehensiveness and clarity	• Specify that the infosheet includes generic recommendations and is not study-specific<br>• Tailor the information in the infosheet to the audience: where applicable, align recommendations with institutional or funder policies, and link to existing support infrastructure (e.g., institutional core facilities)<br>• Where applicable, provide general information beyond specific practices (e.g., in which registry(-ies) should a trial be registered?)
Structure and layout	• Carefully consider how to structure the infosheet (e.g., aggregate resources across all practices in a single place, avoid placing the abbreviations table at the bottom, so that it is not missed)<br>• Arrange practices in a meaningful way (e.g., according to where they are to be implemented, or guidelines vs. regulations)<br>• Use hyperlinks to other sources to provide additional information<br>• Limit the infosheet to 1 page<br>• Consider using a landscape layout for better readability
Language	• Provide an abbreviations table if using acronyms

Infosheet

2 Screening criteria and characteristics of trials and investigators

Supplement 2 Table 1: Characteristics of included trials and investigators.

**Investigator characteristics**	**N = 92**
Contact email found in registry[1]	54 (59%)
**Trial characteristics**	**N = 155**
Registry	
ClinicalTrials.gov	140 (90%)
DRKS	15 (9.7%)
EUCTR[2]	28 (18%)
Completion status	
Completed	129 (83%)
Terminated	12 (7.7%)
Unknown	14 (9.0%)
Completion year	
2014	38 (25%)
2015	50 (32%)
2016	40 (26%)
2017	27 (17%)
**Trial transparency practices included in report cards**	
Prospective registration	94 (61%)[3]
ClinicalTrials.gov	90 (64%)
DRKS	4 (27%)
EUCTR[4]	27 (96%)
Summary results in registry	16 (10%)[3]
ClinicalTrials.gov	16 (11%)
DRKS	0 (0%)
EUCTR	20 (71%)
Summary results in registry within 1 year of completion	8 (5.2%)
ClinicalTrials.gov	8 (5.7%)
DRKS	0 (0%)
Results published in journal article	102 (66%)
Results published in journal article within 2 years of completion	54 (53%)
Trial registration number in publication abstract	34 (33%)
Trial registration number in publication main body	58 (57%)
Publication open access[5]	68 (67%)
Publication linked in registration	53 (52%)[3]
ClinicalTrials.gov	47 (53%)
DRKS	6 (43%)
**Trials eligible for each transparency practice**	
Summary results in registry	**N = 155**
Completed prior to launch	16 (10%)
Not completed prior to launch	139 (90%)
Publication linked in registration[6]	**N = 102**
Completed prior to launch	53 (52%)
Not completed prior to launch	49 (48%)
Publication open access[7]	**N = 100**
Completed prior to launch	68 (68%)
Not completed prior to launch	32 (32%)

[1] We checked both the current (as of 19 May 2022) and historical versions of registrations.
[2] EUCTR was not used as a primary registry data source. Hence, included EUCTR trials reflect cross-registered trials. within the 155 trials with a registration in ClinicalTrials.gov or DRKS.
[3] Limited to trials registered in ClinicalTrials.gov or DRKS, the primary registry data sources in this study. EUCTR is not included.
[4] Prospective registration was assessed based on dates available in the registry. However, in principle all EUCTR trials should be prospectively registered, as registration is linked to regulatory approval by national competent authorities.
[5] Open access includes gold, hybrid, bronze, and green.
[6] Limited to trials with a publication.
[7] Limited to trials with a publication and a publisher permission to self-archive the paper in an institutional repository. For two closed access publications, we could not determine whether such a permission was available.

Supplement 2 Table 2: Trial and investigator inclusion criteria.

Timepoint	Criteria
Prior	Included the Charité as the sponsor, responsible party, or host of the principal investigator
Prior	Study completion date between 2014 or after 2017, study status considered as complete, and interventional
Prior	One or more investigator with email
Post	One or more investigator with successfully delivered email and no auto-reply indicating extended leave throughout our study timeframe

Trial

Timepoint	Criteria
Prior	In ClinicalTrials.gov responsible party (if this included principal investigator), overall study officials (principal investigator, study director, study chair), and central contact person and backup, or in DRKS primary sponsor and contacts for scientific and public queries
Prior	One or more emails found in registry or Google search
Post	Not all emails were undeliverable and no auto-replies indicated extended leave throughout our study timeframe

Investigator

3 Survey administration and detailed results

Response timeline across the survey fielding period

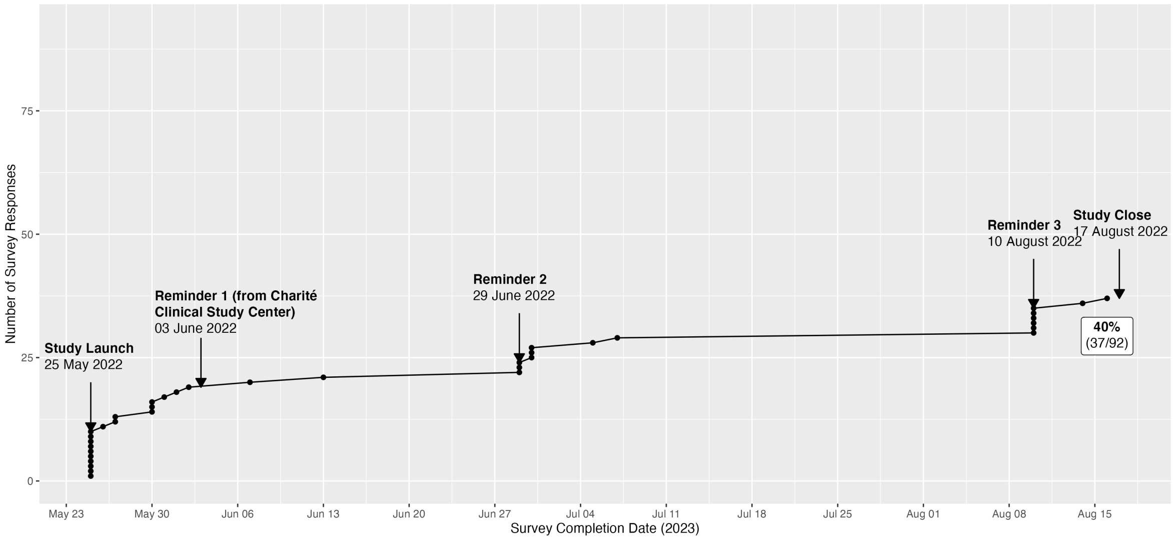

Supplement 3 Figure 1: Timelines of survey responses and key survey dates. The first reminder was sent on behalf of the Clinical Study Center and prompted investigators to review the initial invitation email but did not contain a link to the survey or any other materials. Reminders 2 and 3 both included a link to the survey as well as the report card(s) and infosheet as attachments.

Survey respondents roles

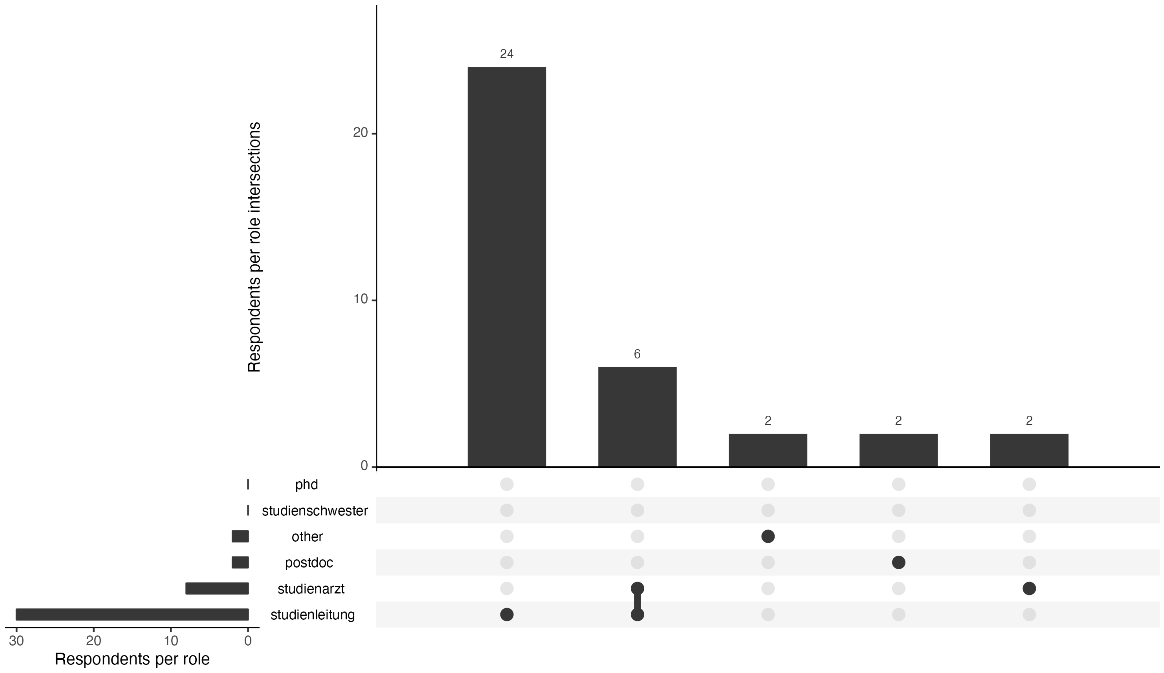

Supplement 3 Figure 2: Survey respondents self-reported roles in trials

The majority of respondents self-reported as study leads (n = 24, 65%), while an additional 6 (16%) self-reported as both study leads and doctors. The remaining respondents identified as either doctors, postdocs, or another position (per group, n = 2, 5.4%).

Themes of comments provided by respondents

Supplement 3 Table 1: Summary of comments from the survey and emails.

**Suggestions for improvement on the materials**	**Detail**
Scope	• Consider including all results publications associated with a trial<br>• Consider expanding report cards to other types of trials<br>• Interest in further information on required documentation for different types of studies<br>• Simplify and focus on essential aspects
Visual design	More professional graphics and design of the report cards and infosheet
Implementation	Send report cards at regular intervals across a study lifecycle
Accessibility	Make the survey bilingual
Times for results reporting	Use event-based times instead of fixed times for results reporting

Challenges related to implementing transparency	Detail
Institutional barriers	• Management and conduct of clinical trials characterized by obstructive, time-consuming institutional processes<br>• Report cards seen as additional administrative bureaucracy, which takes time and resources away from other things<br>• Insufficient protected time for publishing
Legitimacy	Unclear authority of the research team to evaluate studies
Diffusion of responsibility	• Limited control over transparency practices due to journal guidelines<br>• Concern about non-reporting of summary results before publication in a journal
Lack of clarity on practices	• Perception that in case of multiple registrations of a trial, practices can be implemented in only one registry<br>• Focus on legal requirements<br>• Publication abstract or linked publication instead of summary results
Technical difficulties	Technical challenges accessing/managing registry entries

Trial investigator corrections to the report card

Investigators had the opportunity to provide corrections to their report card. Respondents suggested 18 corrections on 11 trials across the survey and emails. In our manual review, we found that many were in fact not corrections but rather incorrect understandings of the practices or additional information on the trials beyond the scope of the report card. This, for example, included links to additional results publications, or information that a publication was open access when in fact it was only accessible via an institutional subscription. After limiting these to valid corrections, 5 corrections regarding 4 trials remained: 1 missed earlier results publication, 1 missed results publication, 1 missed cross-registration in EUCTR, and 2 missed publication links in DRKS.

Subgroup analyses of survey responses

We conducted two post-hoc subgroup analyses. Supplement 3 Figure 3 shows means and 95% confidence intervals for agreement (i.e., Likert-type response item) with each of eight statements about the report card and materials, across all respondents as well as the for the subgroup analyses.

Study role: We explored perceptions of the report card and infosheet across different study roles. We did not observe a meaningful difference between study leads and doctors (n = 32) versus other respondents (n = 5), who were a smaller group with a wide spread of responses.

Review of report card and infosheet: A total of 3 respondents reported not reviewing the report card and infosheet prior to starting the survey and were asked to do so prior to continuing the survey. Per our prespecified inclusion criteria, we did not exclude these responses. However, response time did not significantly differ between those who were and were not asked to review the materials ($\Delta M = 328.31$, 95% CI $[-373.73, 1,030.35]$, $t(2.58) = 1.63$, $p = .215$), which suggests that they did not pause the survey to review the materials. We therefore conducted a post-hoc subgroup analysis. Perceptions of the report card and infosheet were substantially lower among those who reported not reviewing the materials prior to starting the survey: all Likert responses across all 8 items for all 3 respondents were the most negative option, and their free-text responses also included negative comments.

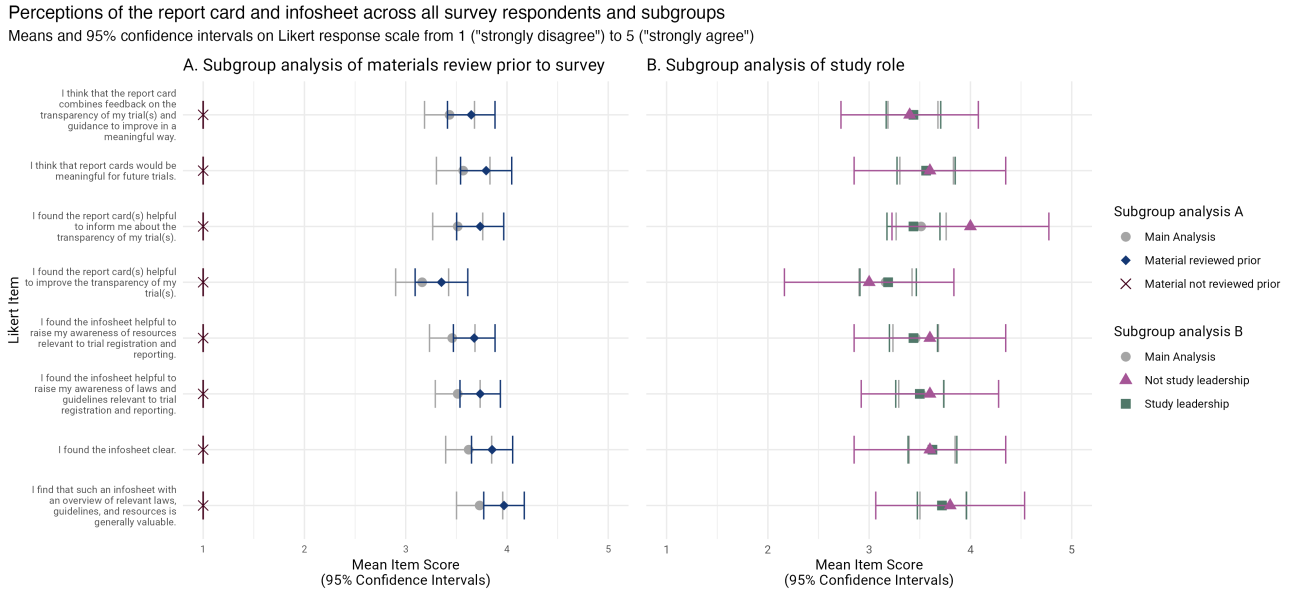

Supplement 3 Figure 3: Perceptions of the report card and infosheet across all survey respondents and subgroups

4 Steps to generate individualized trial transparency report cards

Main steps to prepare a trial transparency dataset for use with the report cards

Supplement 4 Table 1: Steps to create a trial transparency dataset for use with the report card tool.

Step	Manual or automated	Notes
Generate cohort of trials at the level of an institution	Semi-automated	Trials associated with a given institution may be extracted from the registry using automated methods. However, a manual review of affiliations is still required.
Find results publication(s) associated with trials	Semi-automated	Linked results publications may be extracted from the registry using automated methods. However, a manual search for results publications is required since: a) linked publications in the registry are not always results publications (e.g., protocols, reviews), and b) results publications are often not linked in the registration.
Assess transparency of trials (e.g., prospective registration)	Automated to semi-automated	Transparency practices can be evaluated using code, depending on the registry.
Data validation (e.g., cross-registrations)	Manual	Some elements of the report card may require additional manual validation, such as for example suspected cross-registrations.

Additionally, the following code repositories provide more detailed information (e.g., codebook) on the automated steps used to generate components for the report cards:

- https://github.com/quest-bih/trackvalue
- https://github.com/maia-sh/intovalue-data

Automated pipeline to generate individualized report cards

The pipeline is based on a report card template that includes all possible outcomes of a trial's performance represented as layers. The report card template is exported as SVG, which is an image format based on XML. This means that the report card template can be accessed and modified using code. A custom-made Python script selects the correct layers to include for each trial based on a trial transparency dataset. Each report card is automatically exported as PDF.

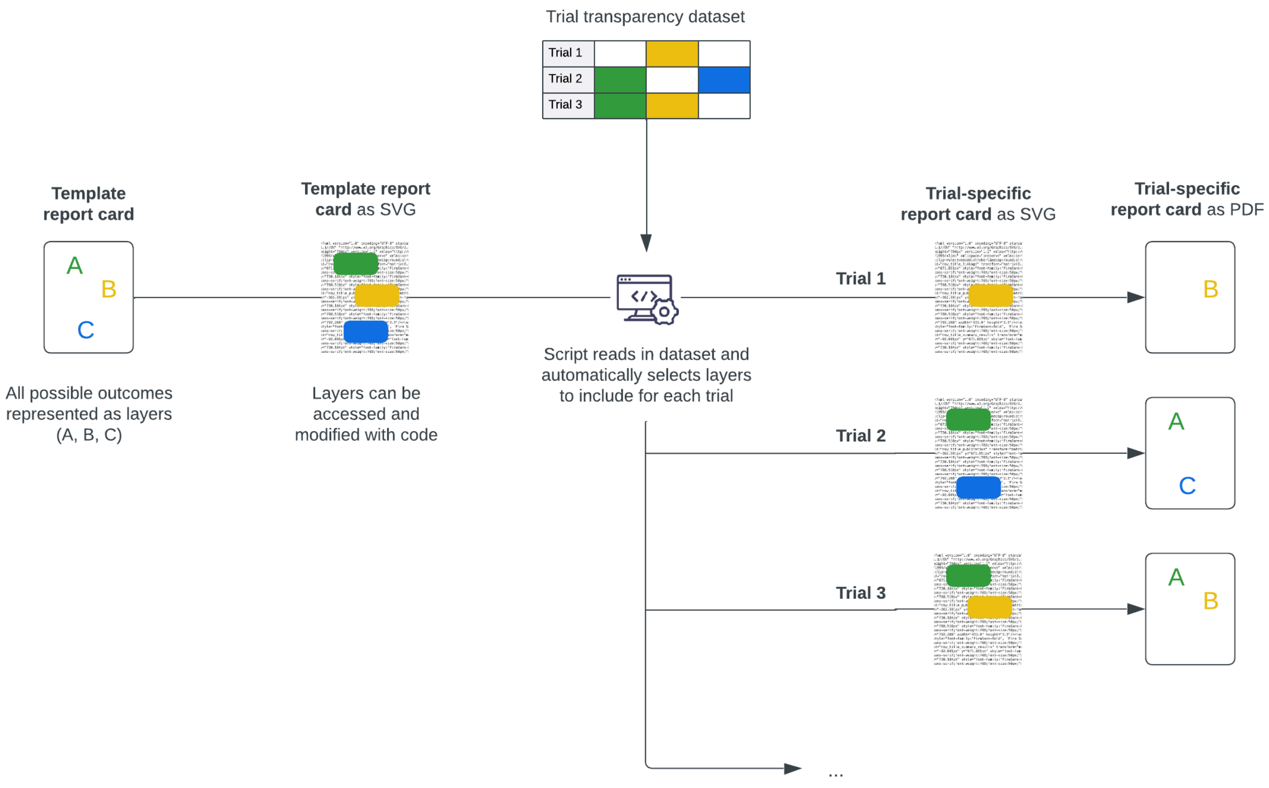

Supplement 4 Figure 1: Automated pipeline to generate individualized report cards based on a trial transparency dataset. PDF: Portable Document Format; SVG: Scalable Vector Graphics.

5 Methods for manual validation of open access status of publications and cross-registration in the EUCTR

The report cards developed for this study included references to any known EUCTR cross-registration (additional registrations of a single trial in a different or same registry). To best ensure that our dataset accurately captured cross-registrations, we manually validated suspected cross-registrations based on the primary registration, and for trials with published results, in the full-text as well as PubMed metadata and abstract. A detailed protocol of these cross-registration checks is available at https://osf.io/mjpzh.

The report cards also called for action on open access (OA) and thus required an accurate OA status of publications. The OA status of publications was determined by querying the Unpaywall API using UnpaywallR. Publications were considered as OA if the peer-reviewed version was openly accessible (without fees or log-in) on either the journal website or a repository. Social networking platforms for researchers (e.g., ResearchGate) or personal websites were not considered as long-term openly accessible venues. Unpaywall has been reported to provide a conservative estimate of the actual percentage of OA in the literature [65], and changes in a publication's OA status are reflected in Unpaywall with a delay. Therefore, we performed manual checks throughout the study of the OA status of publications that were marked as closed in Unpaywall. Manual checks of the OA status of publications were limited to the following study time points: launch, 3 months post-launch, and 12 months post-launch. Any corrections in OA status based on the manual check performed at launch were also applied to the pre-intervention study point for a given publication. Moreover, any new publications shared by investigators were manually checked before sending updated report cards; in these cases, this OA status was also applied to previous study time points. A detailed protocol of these OA checks is available at https://osf.io/3upgy.

6 Reporting checklists

CROSS Checklist for Reporting Of Survey Studies

Sharma, A., Minh Duc, N. T., Luu Lam Thang, T., Nam, N. H., Ng, S. J., Abbas, K. S., Huy, N. T., Marušić, A., Paul, C. L., Kwok, J., Karbwang, J., de Waure, C., Drummond, F. J., Kizawa, Y., Taal, E., Vermeulen, J., Lee, G. H. M., Gyedu, A., To, K. G., … Karamouzian, M. (2021). A Consensus-Based Checklist for Reporting of Survey Studies (CROSS). Journal of General Internal Medicine, 36(10), 3179–3187. https://doi.org/10.1007/s11606-021-06737-1

**Section/topic**	**Item**	**Item description**	**Reported in section *[name]***
**Title and abstract**			
Title and abstract	1a	State the word “survey” along with a commonly used term in title or abstract to introduce the study’s design.	Abstract
	1b	Provide an informative summary in the abstract, covering background, objectives, methods, findings/results, interpretation/discussion, and conclusions.	Abstract
**Introduction**			
Background	2	Provide a background about the rationale of study, what has been previously done, and why this survey is needed.	Introduction, paragraphs 1-4
Purpose/aim	3	Identify specific purposes, aims, goals, or objectives of the study.	Introduction, paragraphs 5-6
**Methods**			
Study design	4	Specify the study design in the methods section with a commonly used term (e.g., cross-sectional or longitudinal).	Methods/Feasibility study
	5a	Describe the questionnaire (e.g., number of sections, number of questions, number and names of instruments used).	Methods/Feasibility study/Materials/Questionnaire development and pretesting;<br><br>OSF project (https://osf.io/stnp5/)
Data collection methods	5b	Describe all questionnaire instruments that were used in the survey to measure particular concepts. Report target population, reported validity and reliability information, scoring/classification procedure, and reference links (if any).	NA
	5c	Provide information on pretesting of the questionnaire, if performed (in the article or in an online supplement). Report the method of pretesting, number of times questionnaire was pre-tested, number and demographics of participants used for pretesting, and the level of similarity of demographics between pre-testing participants and sample population.	Supplement S1;<br><br>Methods/Feasibility study/Materials/Questionnaire development and pretesting;<br><br>OSF project (https://osf.io/stnp5/)
	5d	Questionnaire if possible, should be fully provided (in the article, or as appendices or as an online supplement).	OSF project (https://osf.io/stnp5/)
Sample characteristics	6a	Describe the study population (i.e., background, locations, eligibility criteria for participant inclusion in survey, exclusion criteria).	Methods/Feasibility study/Sample development;<br><br>Supplement S2

	6b	Describe the sampling techniques used (e.g., single stage or multistage sampling, simple random sampling, stratified sampling, cluster sampling, convenience sampling). Specify the locations of sample participants whenever clustered sampling was applied.	Methods/Feasibility study/Sample development
	6c	Provide information on sample size, along with details of sample size calculation.	Methods/Feasibility study/Sample development
	6d	Describe how representative the sample is of the study population (or target population if possible), particularly for population-based surveys.	Methods/Feasibility study/Sample development
Survey administration	7a	Provide information on modes of questionnaire administration, including the type and number of contacts, the location where the survey was conducted (e.g., outpatient room or by use of online tools, such as SurveyMonkey).	Methods/Feasibility study/Materials/Administration of intervention and survey;<br>Methods/Feasibility study/Materials/Questionnaire development and pretesting;<br>Methods/Feasibility study/Sample development/Trial investigators<br>Supplement S3
	7b	Provide information of survey's time frame, such as periods of recruitment, exposure, and follow-up days.	Methods/Feasibility study/Materials/Administration of intervention and survey
	7c	Provide information on the entry process:<br>–>For non-web-based surveys, provide approaches to minimize human error in data entry.<br>–>For web-based surveys, provide approaches to prevent "multiple participation" of participants.	Methods/Feasibility study/Analysis/Analysis of perceptions of the report card and infosheet
Study preparation	8	Describe any preparation process before conducting the survey (e.g., interviewers' training process, advertising the survey).	Supplement S1
Ethical considerations	9a	Provide information on ethical approval for the survey if obtained, including informed consent, institutional review board [IRB] approval, Helsinki declaration, and good clinical practice [GCP] declaration (as appropriate).	Methods/Feasibility study/Ethical approval and study protocol
	9b	Provide information about survey anonymity and confidentiality and describe what mechanisms were used to protect unauthorized access.	Methods/Feasibility study/Materials/Questionnaire development and pretesting;<br>Methods/Feasibility study/Materials/Administration of intervention and survey<br>Methods/Feasibility study/Analysis/Analysis of perceptions of the

			report card and infosheet
Statistical analysis	10a	Describe statistical methods and analytical approach. Report the statistical software that was used for data analysis.	Methods/Feasibility study/Analysis/Analysis of perceptions of the report card and infosheet
	10b	Report any modification of variables used in the analysis, along with reference (if available).	Protocol deviations (see OSF project: https://osf.io/stnp5/)
	10c	Report details about how missing data was handled. Include rate of missing items, missing data mechanism (i.e., missing completely at random [MCAR], missing at random [MAR] or missing not at random [MNAR]) and methods used to deal with missing data (e.g., multiple imputation).	Methods/Feasibility study/Analysis/Analysis of perceptions of the report card and infosheet
	10d	State how non-response error was addressed.	Methods/Feasibility study/Analysis/Analysis of perceptions of the report card and infosheet
	10e	For longitudinal surveys, state how loss to follow-up was addressed.	NA
	10f	Indicate whether any methods such as weighting of items or propensity scores have been used to adjust for non-representativeness of the sample.	NA
	10g	Describe any sensitivity analysis conducted.	Methods/Feasibility study/Analysis/Analysis of perceptions of the report card and infosheet
**Results**			
Respondent characteristics	11a	Report numbers of individuals at each stage of the study. Consider using a flow diagram, if possible.	Figure 2
	11b	Provide reasons for non-participation at each stage, if possible.	Figure 2<br>Results/Feasibility study/Sample characteristics<br>Results/Feasibility study/Survey
	11c	Report response rate, present the definition of response rate or the formula used to calculate response rate.	Results/Feasibility study/Survey;<br>Figure 2;<br>Supplement S3
	11d	Provide information to define how unique visitors are determined. Report number of unique visitors along with relevant proportions (e.g., view proportion, participation proportion, completion proportion).	Methods/Feasibility study/Analysis/Analysis of perceptions of the report card and infosheet
Descriptive results	12	Provide characteristics of study participants, as well as information on potential confounders and assessed outcomes.	Results/Feasibility study/Sample characteristics;<br>Results/Feasibility study/Survey;<br>Supplements S2-S3

Main findings	13a	Give unadjusted estimates and, if applicable, confounder-adjusted estimates along with 95% confidence intervals and p-values.	Figure 3;<br>Supplement S3;<br>Results/Feasibility study/Survey
	13b	For multivariable analysis, provide information on the model building process, model fit statistics, and model assumptions (as appropriate).	NA
	13c	Provide details about any sensitivity analysis performed. If there are considerable amount of missing data, report sensitivity analyses comparing the results of complete cases with that of the imputed dataset (if possible).	Results/Feasibility study/Survey;<br>Supplement S3
**Discussion**			
Limitations	14	Discuss the limitations of the study, considering sources of potential biases and imprecisions, such as non-representativeness of sample, study design, important uncontrolled confounders.	Discussion/Strengths, limitations, and challenges
Interpretations	15	Give a cautious overall interpretation of results, based on potential biases and imprecisions and suggest areas for future research.	Discussion/Summary of findings/Perceptions of the report card and infosheet;<br>Discussion/Implications and future work
Generalizability	16	Discuss the external validity of the results.	Discussion/Research in context
**Other sections**			
Role of funding source	17	State whether any funding organization has had any roles in the survey's design, implementation, and analysis.	Funding
Conflict of interest	18	Declare any potential conflict of interest.	Declarations
Acknowledgement s	19	Provide names of organizations/persons that are acknowledged along with their contribution to the research.	Acknowledgements

CONSORT Checklist for Pilot and Feasibility Trials

Eldridge, S. M., Chan, C. L., Campbell, M. J., Bond, C. M., Hopewell, S., Thabane, L., Lancaster, G. A., Altman, D., Bretz, F., Campbell, M., Cobo, E., Craig, P., Davidson, P., Groves, T., Gumedze, F., Hewison, J., Hirst, A., Hoddinott, P., Lamb, S. E., … on behalf of the PAFS consensus group. (2016). CONSORT 2010 statement: Extension to randomised pilot and feasibility trials. Pilot and Feasibility Studies, 2(1), 64. https://doi.org/10.1186/s40814-016-0105-8.

Table adapted from:
https://figshare.com/articles/journal_contribution/CONSORT_2010_checklist_of_information_to_include_when_reporting_a_pilot_or_feasibility_trial_/21519771.

Section/Topic	Item No	Checklist item	Reported in section *[name]*
**Title and abstract**			
	1a	Identification as a pilot or feasibility randomised trial in the title	Title
	1b	Structured summary of pilot trial design, methods, results, and conclusions (for specific guidance see CONSORT abstract extension for pilot trials)	Abstract
**Introduction**			
Background and objectives	2a	Scientific background and explanation of rationale for future definitive trial, and reasons for randomised pilot trial	Introduction, paragraphs 1-4
	2b	Specific objectives or research questions for pilot trial	Introduction, paragraphs 5-6
**Methods**			
Trial design	3a	Description of pilot trial design (such as parallel, factorial) including allocation ratio	Methods/Feasibility study
	3b	Important changes to methods after pilot trial commencement (such as eligibility criteria), with reasons	Protocol deviations (see OSF project: https://osf.io/stnp5/)
Participants	4a	Eligibility criteria for participants	Methods/Feasibility study/Sample development;<br><br>Supplement S2
	4b	Settings and locations where the data were collected	Methods/Feasibility study/Materials/Administration of intervention and survey
	4c	How participants were identified and consented	Methods/Feasibility study/Sample development/Trial investigators;<br><br>Methods/Feasibility study/Materials/Administration of intervention and survey<br><br>OSF project: https://osf.io/stnp5/
Interventions	5	The interventions for each group with sufficient details to allow replication, including how and when they were actually administered	Methods/Feasibility study/Materials/Administration of intervention and survey;

			Methods/Tools: report cards and infosheet;<br><br>OSF project: https://osf.io/stnp5/
Outcomes	6a	Completely defined prespecified assessments or measurements to address each pilot trial objective specified in 2b, including how and when they were assessed	Methods/Feasibility study/Analysis/Analysis of improvement in transparency practices<br><br>Preregistration in OSF: https://osf.io/stnp5/
	6b	Any changes to pilot trial assessments or measurements after the pilot trial commenced, with reasons	Protocol deviations (see OSF project: https://osf.io/stnp5/)
	6c	If applicable, prespecified criteria used to judge whether, or how, to proceed with future definitive trial	NA
Sample size	7a	Rationale for numbers in the pilot trial	Methods/Feasibility study;<br><br>Methods/Feasibility study/Sample development
	7b	When applicable, explanation of any interim analyses and stopping guidelines	NA
Randomisation:			
Sequence generation	8a	Method used to generate the random allocation sequence	NA
	8b	Type of randomisation(s); details of any restriction (such as blocking and block size)	NA
Allocation Concealment mechanism	9	Mechanism used to implement the random allocation sequence (such as sequentially numbered containers), describing any steps taken to conceal the sequence until interventions were assigned	NA
Implementation	10	Who generated the random allocation sequence, who enrolled participants, and who assigned participants to interventions	NA
Blinding	11a	If done, who was blinded after assignment to interventions (for example, participants, care providers, those assessing outcomes) and how	NA
	11b	If relevant, description of the similarity of interventions	NA
Statistical methods	12	Methods used to address each pilot trial objective whether qualitative or quantitative	Methods/Feasibility study/Analysis/Analysis of improvement in transparency practices
**Results**			
Participant flow (a diagram is strongly recommended)	13a	For each group, the numbers of participants who were approached and/or assessed for eligibility, randomly assigned, received intended treatment, and were assessed for each objective	Figure 2
	13b	For each group, losses and exclusions after randomisation, together with reasons	Figure 2;<br><br>Results/Feasibility study/Sample characteristics

Recruitment	14a	Dates defining the periods of recruitment and follow-up	Supplement S3
	14b	Why the pilot trial ended or was stopped	NA
Baseline data	15	A table showing baseline demographic and clinical characteristics for each group	Supplement S2
Numbers analysed	16	For each objective, number of participants (denominator) included in each analysis. If relevant, these numbers<br>should be by randomised group	Figure 2
Outcomes and estimation	17	For each objective, results including expressions of uncertainty (such as 95% confidence interval) for any estimates. If relevant, these results should be by randomised group	Figure 4;<br><br>Results/Feasibility study/Improvement in transparency practices
Ancillary analyses	18	Results of any other analyses performed that could be used to inform the future definitive trial	Supplement S3
Harms	19	All important harms or unintended effects in each group (for specific guidance see CONSORT for harms)	NA
	19a	If relevant, other important unintended consequences	NA
**Discussion**			
Limitations	20	Pilot trial limitations, addressing sources of potential bias and remaining uncertainty about feasibility	Discussion/Strengths, limitations, and challenges
Generalisability	21	Generalisability (applicability) of pilot trial methods and findings to future definitive trial and other studies	Discussion/Scaling beyond transparency in clinical trials;<br><br>Discussion/Implications and future work
Interpretation	22	Interpretation consistent with pilot trial objectives and findings, balancing potential benefits and harms, and considering other relevant evidence	Discussion/Summary of findings/Transparency improvements in practice;<br><br>Discussion/Research in context
	22a	Implications for progression from pilot to future definitive trial, including any proposed amendments	Discussion/Implications and future work
**Other information**			
Registration	23	Registration number for pilot trial and name of trial registry	NA
Protocol	24	Where the pilot trial protocol can be accessed, if available	Methods/Feasibility study/Ethical approval and study protocol
Funding	25	Sources of funding and other support (such as supply of drugs), role of funders	Funding
	26	Ethical approval or approval by research review committee, confirmed with reference number	Methods/Feasibility study/Ethical approval and study protocol
